# Septic shock-3 vs 2: an analysis of the ALBIOS study

**DOI:** 10.1186/s13054-018-2169-8

**Published:** 2018-09-27

**Authors:** Francesco Vasques, Eleonora Duscio, Federica Romitti, Iacopo Pasticci, Pietro Caironi, Jennifer Meessen, Roberto Latini, Massimo Cressoni, Luigi Camporota, Antonio Pesenti, Roberto Fumagalli, Michael Quintel, Luciano Gattinoni

**Affiliations:** 1 Department of Anesthesiology, Emergency and Intensive Care Medicine, University of Göttingen, Robert-Koch-Straße, 40, 37075 Göttingen, Germany; 2 Department of Anesthesia and Critical Care, Azienda-Ospedaliero Universitaria S. Luigi Gonzaga, Orbassano, Italy; 3 Dipartimento di Oncologia, Università degli Studi di Torino, Turin, Italy; 4 Department of Cardiovascular Research, IRCCS - Istituto di Ricerche Farmacologiche “Mario Negri”, Milan, Italy; 5 Dipartimento di Scienze della Salute, Università degli Studi di Milano, Milan, Italy; 6 Department of Adult Critical Care, Guy’s and St Thomas’ NHS Foundation Trust, King’s Health Partners, and Division of Asthma, Allergy and Lung Biology, King’s College London, London, UK; 7 Dipartimento di Anestesia, Rianimazione ed Emergenza Urgenza, Fondazione IRCCS Ca’ Granda - Ospedale Maggiore Policlinico, Milan, Italy; 8 Dipartimento di Fisiopatologia Medico-Chirurgica e dei Trapianti, Università degli Studi di Milano, Milan, Italy; 9 University of Milan-Bicocca, School of Medicine and Surgery, Monza, Italy

**Keywords:** Septic shock, Shock-3, Sepsis, Albumin, Crystalloids, ALBIOS

## Abstract

**Background:**

A reanalysis of the ALBIOS trial suggested that patients with septic shock - defined by vasopressor-dependent hypotension in the presence of severe sepsis (Shock-2) - had a survival benefit when treated with albumin. The new septic shock definition (Shock-3) added the criterion of a lactate threshold of 2 mmol/L. We investigated how the populations defined according to Shock-2 and Shock-3 differed and whether the albumin benefit would be confirmed.

**Methods:**

This is a retrospective analysis of the ALBIOS study, a randomized controlled study conducted between 2008 and 2012 in 100 intensive care units in Italy comparing the administration of 20% albumin and crystalloids versus crystalloids alone in patients with severe sepsis or septic shock. We analyzed data from 1741 patients from ALBIOS with serum lactate measurement available at baseline. We compared group size, physiological variables and 90-day mortality between patients defined by Shock-2 and Shock-3 and between the albumin and crystalloid treatment groups.

**Results:**

We compared the Shock-2 and the Shock-3 definitions and the albumin and crystalloid treatment groups in terms of group size and physiological, laboratory and outcome variables. The Shock-3 definition reduced the population with shock by 34%. The Shock-3 group had higher lactate (*p* < 0.001), greater resuscitation-fluid requirement (*p* = 0.014), higher Simplified Acute Physiology Score II (*p* < 0.001) and Sepsis-related Organ Failure Assessment scores (*p* = 0.022), lower platelet count (*p* = 0.002) and higher 90-day mortality (46.7% vs 51.9%; *p* = 0.031). Albumin decreased mortality in Shock-2 patients compared to crystalloids (43.5% vs 49.9%; 12.6% relative risk reduction; *p* = 0.04). In patients defined by Shock-3 a similar benefit was observed for albumin with a 11.3% relative risk reduction (48.7% vs 54.9%; 11.3% relative risk reduction; *p* = 0.22).

**Conclusions:**

The Sepsis-3 definition reduced the size of the population with shock and showed a similar effect size in the benefits of albumin. The Shock-3 criteria will markedly slow patients’ recruitment rates, in view of testing albumin in septic shock.

**Trial registration:**

ClinicalTrials.gov, number NCT00707122. Registered on 30 June 2008.

## Background

Septic shock is a major challenge in intensive care and its treatment remains elusive despite decades of basic, translational and clinical research. Given that sepsis is a syndrome rather than a disease, the individual response to treatment may be heavily confounded by its underlying etiology and pathophysiologic phenotype, which represent an unavoidable source of heterogeneity in patients with sepsis. An additional source of heterogeneity however, derives from the severity of sepsis. Indeed, various clinical definitions of septic shock identify patients with different severity and mortality risk, depending on the criteria they employ. Historically, at the beginning of the randomized clinical studies era, the inclusion criteria were mainly based on the authors’ initiative. Therefore, efforts have been made to select criteria that identify a more homogeneous subset of septic patients with a greater mortality risk than the one associated to sepsis alone. A consensus conference in 1991 defined septic shock as “sepsis-induced hypotension [persisting] despite adequate fluid resuscitation”, which could be further categorized as cardiovascular Sepsis-related Organ Failure Assessment (SOFA) score of 1, 3 or 4 [1–3]. However, the application of similar criteria led to the enrollment of remarkably different populations into clinical trials in different countries, with striking differences in mortality rates [4]. In Italy, the ALBIOS study had an overall 90-day mortality rate of 42.2% [1], similar to the one recorded in Scandinavia (44%) [5] and in France (43%) [6]. However, the mortality was remarkably lower in trials conducted in the USA (32%) [7] or in Australia and New Zealand (18.8%) [8]. These trials highlighted the fact that the “Sepsis 2” criteria for septic shock were inadequate to identify patients with comparable severity and raised the issue of the results of these trials not being generalizable to the “real world” population (i.e., external validity). In 2016, a new consensus definition - in an effort to increase predictive validity - proposed the need for both vasopressor-dependent hypotension and serum lactate greater than 2 mmol/L after adequate fluid resuscitation to define septic shock (Shock-3 definition) [9]. Indeed, the association between serum lactate levels and mortality is one of the oldest and most consistent relationship in intensive care and its inclusion in the definition of shock is based on the clear correlation between excess lactate level and severity of illness, the lactate level being a crude surrogate for cellular and metabolic abnormalities [10].

In this study, we reanalyzed the results from the ALBIOS study [1] - which in 2014 compared the efficacy of the administration of albumin and crystalloids versus crystalloids alone in patients with septic shock - based on the new definition of septic shock. Our aim was to report differences in the populations classified as Shock-2 or Shock-3, highlighting possible advantages and disadvantages of this new definition.

## Methods

This study was a retrospective analysis of data from the ALBIOS study [1]. ALBIOS was a multicenter randomized controlled trial (RCT) conducted between 2008 and 2012 in 100 Italian intensive care units, comparing the administration of 20% albumin and crystalloids versus crystalloids alone from randomization to day 28 or discharge from ICU (whatever came first), in the treatment of 1818 adult patients with severe sepsis or septic shock (eligibility criteria available in the online supplement of the ALBIOS trial [1]). In Table 1, we summarize the criteria we used for the septic shock definition in the ALBIOS study and the one used in the present study. As shown, the differences were related to the availability of lactate levels at baseline and the lactate threshold. Accordingly, we compared the patient subpopulations defined on the basis of Shock-2 (1098 patients) and Shock-3 (721 patients) criteria in terms of physiological, gas exchange, hemodynamic and outcome variables.

**Table 1:** Patient population

Entry criteria		Septic shock ALBIOS	Shock-2 present study	Shock-3 present study
		Severe sepsis + cardiovascular Sepsis-related Organ Failure Assessment score 3 or 4	As septic shock ALBIOS, in which baseline lactate was available	Shock-2 patients with baseline lactate > 2 mmol/L
Patients	*n*	1135	1098	721
Patients lost at follow up	*n*	14	12	8
Mortality data available	*n*	1121	1086	713

### Statistical analysis

All the analyses were conducted on an intention-to-treat basis. Binary outcomes were compared with the use of the chi-square test and continuous outcomes with the use of the Wilcoxon rank-sum test. Results were reported as mean plus or minus standard deviation, as appropriate. Survival estimates were calculated according to the Kaplan–Meier method and compared using the log-rank test, stratified for Sepsis-2 and Sepsis-3 definitions, to compare the albumin and crystalloid treatments. *p* values < 0.05 were considered significant. We performed all analyses with R software.

## Results

**Fig. 1:**
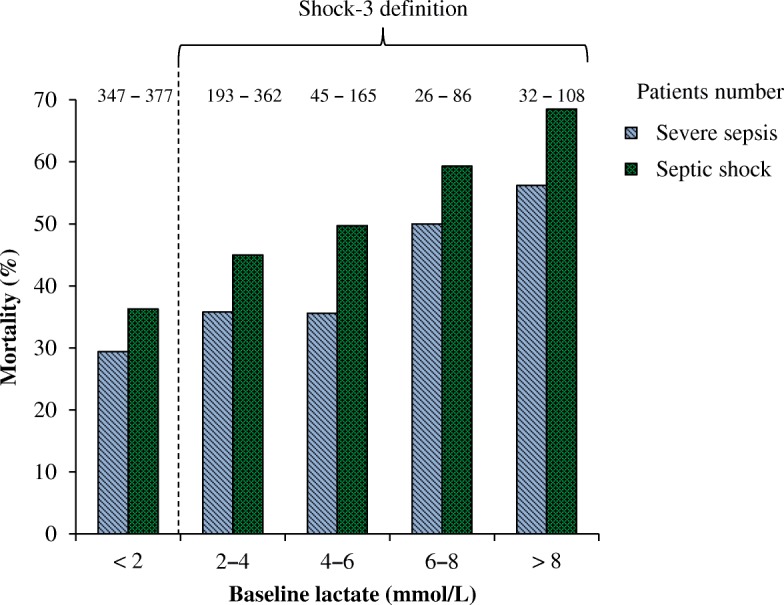
Mortality according to baseline lactate levels in patients with severe sepsis (light bars) or septic shock (dark bars). Chi-square test: sepsis, *p* = 0.008; septic shock, *p* < 0.0001

**Fig. 2:**
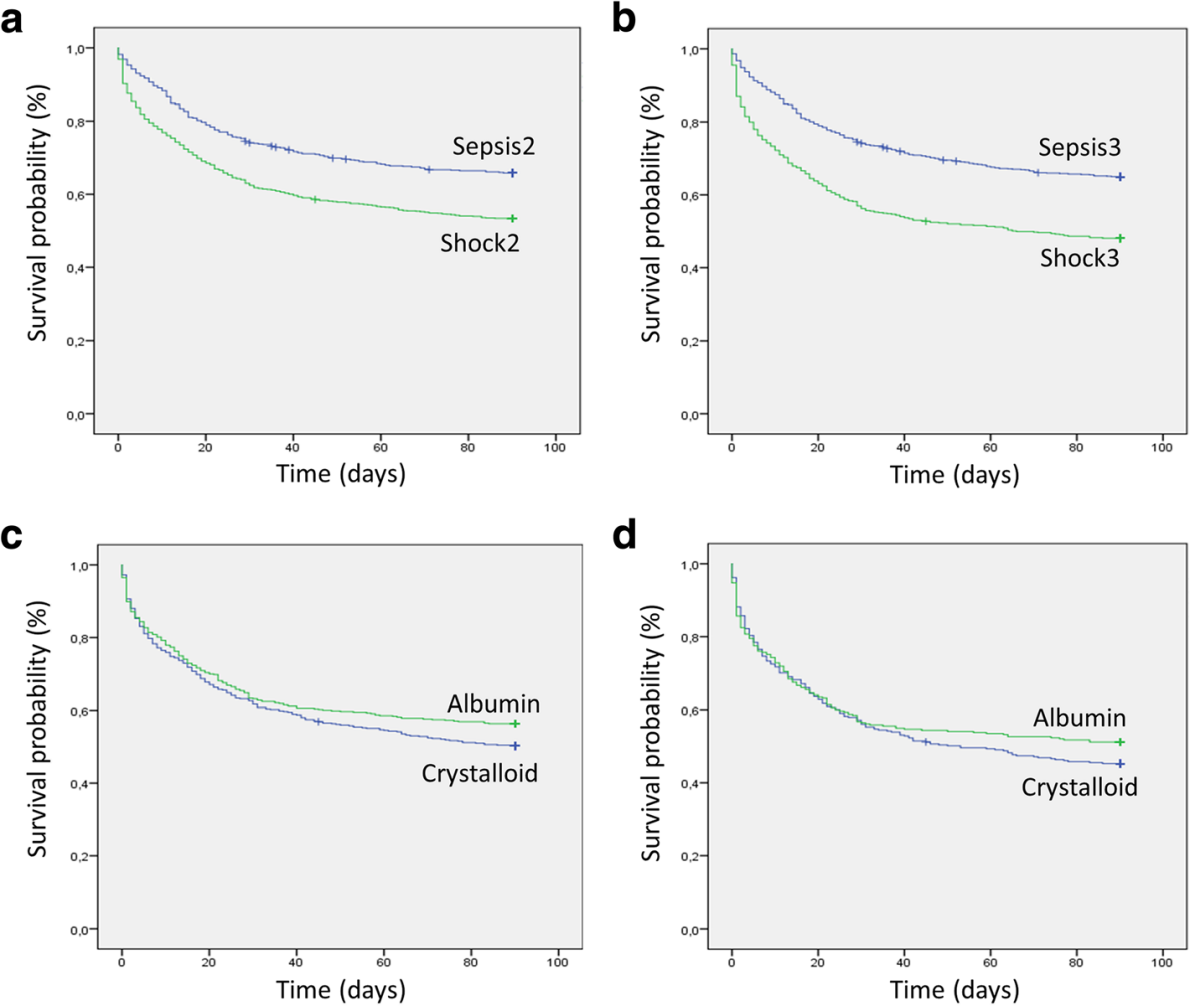
Probability of survival from randomization to day 90. **a** Kaplan–Meier estimates for the probability of survival among patients classified by Sepsis-2 versus Shock-2. Mean survival time 66.7 (95% CI 64.1–69.2) vs 56.4 (95% CI54.1–58.7) days. Log rank test *p* < 0.001. Absolute 90-day mortality 34.1% vs 46.7%, respectively. Chi-square test *p* < 0.001. **b** Kaplan–Meier estimates of Sepsis-3 vs Shock-3. Mean survival time 66.3 (95% CI 64.2–68.4) vs 51.6 (95% CI 48.7–54.5) days. Log rank test *p* < 0.001. Absolute 90-day mortality 35.0% vs 51.9%, respectively. Chi-square *p* < 0.001). **c** Kaplan–Meier estimates for the probability of survival in patients defined by Shock-2, treated with albumin versus crystalloids. Mean survival time 57.9 (95% CI 54.7–61.1) vs 54.9 (95% CI 51.7–58.2). Log rank test *p* = 0.082. Absolute 90-day mortality 43.5% vs 49.9% (absolute risk reduction 6.3%, risk ratio 0.89, 95% CI 0.79–0.99). Chi-square test *p* = 0.04. **d** Kaplan–Meier estimates for the probability of survival in patients defined by Shock-3, treated with albumin versus crystalloids. Mean survival time: 52.9 (95% CI 48.6–57.1) vs 50.4 (95% CI 46.4–54.4). Log rank test *p* = 0.246. Absolute 90-day mortality 48.7% vs 54.9% (absolute risk reduction 6.2%, risk ratio 0.88, 95% CI 0.75–1.02). Chi-square test *p* = 0.11

In Fig. 1, we show the mortality rate as a function of baseline serum lactate level recorded in patients with severe sepsis with (Septic shock, *n* = 1098) or without shock (Severe sepsis, *n* = 643) according to the ALBIOS definition. As shown, mortality increased with increasing baseline lactate, both in patients with septic shock (*p* < 0.0001) and in patients with severe sepsis without shock (*p* = 0.008). In the same figure, we show the Shock-3 population, which includes only patients with baseline serum lactate above 2 mmol/L. As shown, 377 patients (34%) previously classified as having septic shock, no longer met the criteria for shock - based on the lactate criterion (Shock-3 definition). The change in the inclusion criteria increased the group of patients with severe sepsis without shock from 643 to 1020 patients. In Fig. 2a we show the probability of survival according to the Sepsis-2 and Sepsis-3 definitions. Of note, at 90 days, survival was significantly lower in the subgroup of patients with septic shock and higher lactate (Shock-3) than in patients with shock that had been defined according to Sepsis 2 criteria (Shock-2) independent from the lactate level (chi-square test *p* = 0.031).

### Patients with septic shock

In Table 2, we compare several baseline physiological and clinical variables recorded following the Shock-2 versus Shock-3 definition. The application of the new classification decreased the population with septic shock by about 34% and increased its severity. Indeed, besides the higher serum lactate levels dictated by the definition criteria, patients defined by Shock-3 had a significantly higher mortality rate (absolute mortality difference of 5.2%), higher SOFA scores and higher Simplified Acute Physiology (SAPS)-II scores. In addition, they had a significantly lower platelet count, a more positive fluid balance at 6 h and received a larger amount of fluid resuscitation in the first 24 h.

**Table 2:** Patients with septic shock (Shock-2 and Shock-3 definition) treated with albumin or crystalloids

	Shock 2 *N* = 1098			Shock 3 *N* = 721		
	Albumin *N* = 549	Crystalloids *N* = 549	*p* value	Albumin *N* = 346	Crystalloids *N* = 375	*p* value
MAP (mmHg)	70.9 ± 14.4	70.8 ± 14.1	0.958^a^	70.4 ± 14.8	69.0 ± 14.0	0.190^a^
CVP (mmHg)	10.8 ± 5.0	10.8 ± 4.9	0.877^a^	11.0 ± 5.1	10.8 ± 4.9	0.583^a^
HR (bpm)	106.5 ± 22.1	106.8 ± 20.4	0.819^a^	109.8 ± 21.5	109.2 ± 19.8	0.672^a^
SvO_2_ (%)	71.4 ± 11.14	73.3 ± 10.0	0.005^a^	70.7 ± 11.9	73.0 ± 10.2	0.007^a^
PaCO_2_ (mmHg)	39.2 ± 12.4	39.2 ± 9.7	0.960^a^	38.1 ± 11.4	38.67 ± 9.8	0.455
PvCO_2_ (mmHg)	46.7 ± 12.6	46.3 ± 10.3	0.524^a^	45.9 ± 11.8	45.69 ± 10.7	0.833^a^
Noradrenaline (μg/kg/min)	1.9 ± 0.8	1.3 ± 0.6	0.042^a^	1.9 ± 0.9	1.3 ± 0.6	0.126^a^
Patients on noradrenaline	463 (84.3%)	458 (83.4%)	0.682^c^	296 (85.5%)	321 (85.6%)	0.985^c^
Lactate (mmol/L)	3.7 ± 3.2	4.1 ± 3.4	0.040^b^	5.11 ± 3.3	5.31 ± 3.4	0.487^b^
pH	7.40 ± 0.1	7.4 ± 0.1	0.883^a^	7.35 ± 0.1	7.34 ± 0.1	0.597^a^
BE (mmol/L)	−3.9 ± 6.1	−3.7 ± 6.2	0.800^b^	−4.8 ± 6.2	−4.6 ± 6.2	0.937^b^
Albumin (g/L)	23.5 ± 6.3	24.1 ± 6.1	0.138^b^	23.4 ± 6.4	23.9 ± 6.2	0.617^b^
Creatinine (mg/dL)	2.2 ± 1.7	2.1 ± 1.5	0.153^a^	2.3 ± 1.6	2.2 ± 1.5	0.175^a^
Diuresis (ml/h)	70.6 ± 71.1	72.7 ± 76.5	0.610^b^	68.8 ± 73.3	69.2 ± 75.7	0.847^b^
Fluid balance (6 h) (L)	1.2 ± 1.4	1.2 ± 1.7	0.549^b^	1.4 ± 1.5	1.5 ± 1.8	0.918^b^
Fluid input (day 1) (L)	4.8 ± 2.3	4.8 ± 2.3	0.917^b^	5.1 ± 2.4	5.1 ± 2.5	0.710^b^
SOFA	9.3 ± 2.6	9.4 ± 2.8	0.782^b^	9.9 ± 2.7	9.9 ± 2.8	0.906^b^
SAPS II	52.1 ± 17.0	52.7 ± 17.1	0.592^a^	55.6 ± 17.0	55.8 ± 17.1	0.911^a^
Mortality (90 day)	237 (43.6%)	270 (49.8%)	0.039^c^	167 (48.7%)	203 (54.9%)	0.099^c^
WBC	13.4 ± 10.3	13.2 ± 10.8	0.762^a^	12.9 ± 11.0	12.6 ± 11.5	0.644^a^
PLT	176 ± 118	181 ± 131	0.873^b^	156 ± 109.9	164 ± 126.1	0.592^b^

Physiological and outcome variables (mean ± standard deviation or number (percentage)) measured at baseline in patients classified according to the criteria for septic shock adopted in the ALBIOS trial compared to the Shock-3 definition

*MAP* mean arterial pressure, *CVP* central venous pressure, *HR* heart rate, *SvO*_*2*_ central venous saturation, *PaCO*_*2*_ arterial CO_2_ partial pressure, *PvCO*_*2*_ venous CO_2_, partial pressure, *BE* base excess, *SOFA* Sepsis-related Organ Failure Assessment, *SAPS-II* Simplified Acute Physiology Score II, *WBC* white cell count, *PLT* platelet count

^a^Analysis of variance for continuous normally distributed variables

^b^Mann–Whitney test for continuous non-normally distributed variables

^c^Chi-square test for discrete variables

### Patients without shock

The reclassification of 377 patients from the “septic shock” to the “severe sepsis without shock” group increased the size of the latter by nearly 60%. Besides the differences linked to the new criteria (e.g., all 377 patients transferred had, by definition, norepinephrine infusion), all the other statistically significant differences that we found do not appear clinically relevant (mean arterial pressure difference 2 mmHg, central venous pressure difference 0.5 mmHg, lactate difference 0.5 mmol/L and pH difference 0.01, results not shown). Mortality was also similar between the subgroup of patients with sepsis based on the Sepsis-2 and Sepsis-3 definitions (34.7% vs 35.5%).

### Sepsis and albumin

The results of the ALBIOS study indicated that the use of albumin in addition to crystalloids, as compared with the use of crystalloids alone, in patients with severe sepsis or septic shock during their stay in the ICU did not provide a survival benefit at 90 days, despite improvements in hemodynamic variables.

#### Patients defined by Shock-2

In Fig. 2a, b, we compare the survival probability of patients defined by Shock-2 and Shock-3 relative to patients defined by Sepsis-2 and Sepsis-3. As shown, in both cases, survival was significantly higher in patients with sepsis compared to patients with shock. In Fig. 2c, we compare the effect of albumin versus crystalloids in patients defined by Shock-2 and Shock-3. As shown, in the population defined by Shock-2, the mean survival days are not significantly different between albumin and control treated patients (log rank test *p* = 0.08); however, the absolute mortality rate at 90 days is significantly different (43.5% vs 49.9%, absolute risk reduction 6.3%, risk ratio 0.89, 95% CI 0.79–0.99, Chi-square test *p* = 0.04). In patients defined by Shock-3, the mortality rate was 48.7% in the albumin group and 54.9% in the control group (absolute risk reduction 6.2%, risk ratio 0.88, 95% CI 0.75–1.02). The mortality difference, however, was not statistically significant (*p* = 0.11), nor was the mean number of survival days (*p* = 0.246).

## Discussion

The main finding of our study was that Shock-3 criteria applied to the ALBIOS population selected a smaller but more severely ill population affected by higher mortality. The relative risk reduction in mortality observed in septic patients defined by Shock-2 and treated with albumin, compared to those treated with crystalloids, remained similar when applying the Sepsis-3 criteria (i.e., lactate > 2 mmol/L) (12.6% vs 11.3%) but was no longer significant, due to the smaller size of the septic shock group. The relative risk reduction from 12.6 to 11.3% suggests that the proposed effect of albumin, if any, does not depend on lactate level and the shock definition. The introduction of the Sepsis-3 definition of septic shock opens a new scenario concerning the reanalysis of previous RCTs and the design of future trials. On the wave of the enthusiasm for the new definition, some authors - like Russell et al. [11] and ourselves - will reanalyze previous RCT data under the light of the new classification of septic shock, either confirming the previous findings or reporting new ones. Regardless of their results, these studies will probably be considered as a new discovery, as they will be obtained from randomized trials conducted by authoritative experts who provided the new septic shock definition. Indeed, the reanalysis by Russell et al. showed that vasopressin was only effective in less severely ill patients (with lactate ≤ 2 mmol/L; i.e., sepsis according to the new definition) - with an absolute risk reduction of 12.3% (hazard ratio (HR) 0.67; 95% CI 0.46–0.96), while it had no benefit in patients with lactate > 2 mmol/L (absolute risk reduction of 2.%; HR 0.97; 95% CI 0.74–1.27). In our study we found a similar relative risk reduction in mortality.

It would not be surprising to find that the effect size of some of the major RCTs that contributed to guide clinical practice may lead to different conclusions if subjected to reanalysis with the inclusion criteria of Sepsis-3. However, we should consider that any reanalysis of a previous trial using the new criteria is no more than an unplanned, post hoc, subgroup analysis and, as such, does not possess enough scientific rigor to change clinical practice.

The goal of the new definition was primarily to increase homogeneity in the severity and risk of death of patients with septic shock while separating them from the patients with sepsis with a lower mortality risk. Undoubtedly, from this perspective, serum lactate works. Russell et al. analyzing the VASST trial [12] found an increase in 90-day mortality in patients with lactate > 2 mmol/L (from 43.9% of the original group to 55% in patients with the Sepsis-3 definition of septic shock), which is consistent with the difference in the mortality risk demonstrated in the validation study of the Sepsis-3 definition [13]. This new proposed classification may lead to greater homogeneity in severity and associated risk of death across all studies in septic shock. This would increase the external validity of the trial particularly when the reported crude mortality rates in the control group are as disparate as 18.8% in ARISE [8], 33.7% in ProCESS [7] and up to 56.9% at 60 days in the study by Rivers et al. [14]. However, the selection of a more severely ill population may present some drawbacks related to the treatment effect size and the repercussions on power and population sample size. In our analysis we found that the introduction of the 2 mmol/L lactate threshold was associated with an absolute increased risk of 5.2% (relative increase 11%). This figure roughly indicates the order of magnitude of the “attributable mortality” due to the increase in sepsis severity associated with the threshold of 2 mmol/L. These data may be helpful in defining the expected changes in mortality in future trials.

The second issue of the sample size may pose greater problems of the feasibility of new RCTs. Indeed, the number of patients required to be enrolled to show a significant difference in outcome increases as the baseline mortality approaches 50% and the length of the study will be greatly prolonged. As an example, targeting an absolute reduction in mortality of 6% from a baseline expected mortality of 40%, the required sample size would be 2030 patients (alpha 0.05 and beta 0.2). As indicated in Table 3, considering 31 participating centers (median value from previous RCTs) and a recruitment rate of 0.68 patients/unit/month (mean value), a study would be completed in 8 years applying the Sepsis-2 criteria. In contrast, applying Sepsis-3 criteria - higher mortality (50%) and 30% smaller population - the same study would require approximately 12 years.

**Table 3:** Enrolment rate of septic shock patients in recent randomized controlled trials on sepsis

Study	TRISS [5]	SEPSISPAM [6]	ProCESS [7]	ARISE [8]	ALBIOS [1]	VASST [12]
Patients enrolled (*n*)	1005	776	1341	1600	1135	778
Enrollment mid-date	2012	2011	2011	2011	2010	2003
Enrollment period (months)	25	22	74	66	42	57
Centers (*n*)	32	29	31	51	100	27
Enrollment rate (patients/month/center)	1.26	1.22	0.58	0.24	0.27	0.52
Enrollment rate (patients/month)	40.3	35.4	18	12	27	14

## Conclusions

We showed that the Shock-3 definition did not affect the treatment effect size (12.6–11.3%) and that possible benefits of albumin were no longer significant. Shock-3 criteria have the advantage of increasing the external validity of studies by selecting a population with more severe sepsis, though not necessarily more homogeneous. However, it has the disadvantages of greater difficulty in recruitment, duration and cost of the trial. Extreme caution must be taken when interpreting previous clinical trials designed for Sepsis-2 criteria being reanalyzed using the new definition, as the finding will have the limitations of a subgroup analysis and, as such, it should not be considered a rigorous finding fulfilling the requirements to influence or change clinical practice.

## Abbreviations

RCT: Randomized controlled trial
SAPS: Simplified Acute Physiology Score
SOFA: Sepsis-related Organ Failure Assessment
